# Hemodynamics in Shock Patients Assessed by Critical Care Ultrasound and Its Relationship to Outcome: A Prospective Study

**DOI:** 10.1155/2020/5175393

**Published:** 2020-09-15

**Authors:** Tongjuan Zou, Wanhong Yin, Yi Li, Lijing Deng, Ran Zhou, Xiaoting Wang, Yangong Chao, Lina Zhang, Yan Kang, Chinese Critical Ultrasound Study Group (CCUSG)

**Affiliations:** ^1^Department of Critical Care Medicine, West China Hospital/West China School of Medicine, Sichuan University, Chengdu, Sichuan 610041, China; ^2^Department of Critical Care Medicine, Peking Union Medical College Hospital, Peking Union Medical College, Chinese Academy of Medical Sciences, Beijing 100730, China; ^3^Department of Critical Care Medicine, The First Hospital of Tsinghua University, Beijing 100016, China; ^4^Department of Critical Care Medicine, Xiangya Hospital, Central South University, Changsha, Hunan 410008, China; ^5^West China Hospital of Sichuan University, Chengdu 610041, China

## Abstract

**Background:**

Shock is one of the causes of mortality in the intensive care unit (ICU). Traditionally, hemodynamics related to shock have been monitored by broad-spectrum devices with treatment guided by many inaccurate variables to describe the pathophysiological changes. Critical care ultrasound (CCUS) has been widely advocated as a preferred tool to monitor shock patients. The purpose of this study was to analyze and broaden current knowledge of the characteristics of ultrasonic hemodynamic pattern and investigate their relationship to outcome.

**Methods:**

This prospective study of shock patients in CCUS was conducted in 181 adult patients between April 2016 and June 2017 in the Department of Intensive Care Unit of West China Hospital. CCUS was performed within the initial 6 hours after shock patients were enrolled. The demographic and clinical characteristics, ultrasonic pattern of hemodynamics, and outcome were recorded. A stepwise bivariate logistic regression model was established to identify the correlation between ultrasonic variables and the 28-day mortality.

**Results:**

A total of 181 patients with shock were included in our study (male/female: 113/68). The mean age was 58.2 ± 18.0 years; the mean Acute Physiology and Chronic Health Evaluation II (APACHE II score) was 23.7 ± 8.7, and the 28-day mortality was 44.8% (81/181). The details of ultrasonic pattern were well represented, and the multivariate analysis revealed that mitral annular plane systolic excursion (MAPSE), mitral annular peak systolic velocity (S′-MV), tricuspid annular plane systolic excursion (TAPSE), and lung ultrasound score (LUSS) were the independent risk factors for 28-day mortality in our study, as well as APACHE II score, PaO_2_/FiO_2_, and lactate (*p* = 0.047, 0.041, 0.022, 0.002, 0.027, 0.028, and 0.01, respectively).

**Conclusions:**

CCUS exam on admission provided valuable information to describe the pathophysiological changes of shock patients and the mechanism of shock. Several critical variables obtained by CCUS were related to outcome, hence deserving more attention in clinical decision-making. *Trial Registration*. The study was approved by the Ethics Committee of West China Hospital Review Board for human research with the following reference number 201736 and was registered on ClinicalTrials. This trial is registered with NCT03082326 on 3 March 2017 (retrospectively registered).

## 1. Introduction

Shock is one of the most common conditions in the intensive care unit (ICU) affecting one-third of critically ill patients. It reduces oxygen and nutrition's perfusion to the solid organs and is closely associated with increased mortality [1–3]. Most literature has described how hemodynamic monitoring could provide an effective way to identify underlying pathophysiological processes and guide appropriate therapy in shock patients [4, 5]. However, highlighting limitations of previous studies, the current widely used measurements such as SWAN-GANZ and pulse index and continuous cardiac output (PICCO) are invasive and focus on specific indices but cannot visualize the structural abnormality of the heart and lung or the flow abnormalities of the circulatory system [6–9]. In other words, they do not have the exact ability to identify crucial pathophysiological changes.

For over 80 years, the ultrasound has been the classic method for visualization of both organ system structural abnormalities and direction of blood flow. Currently, the critical care ultrasound (CCUS) has been widely advocated as the preferred tool to assess hemodynamics [4, 5, 9], including accurately estimating pathophysiological changes of shock [10–16]. This information, therefore, can be out into protocols to guide shock treatment. However, despite previous recommendations, current protocols are subjective and empirical, without listing specific variables as indicators, such as ejection fraction (EF), mitral annular plane systolic excursion (MAPSE), tricuspid annular plane systolic excursion (TAPSE), mitral or tricuspid annular peak systolic velocity (S′-MV or S′-TV), inferior vena cava (IVC), and lung ultrasound score (LUSS) [17–21].

Previous studies have shown that ultrasound leads to a prompt diagnosis in addition to the reduction in health care costs, hospitalization period, and mortality [16, 17, 21–23]. In our previous retrospective study, we demonstrated that several ultrasonic indicators used on ICU admission have been able to predict the 28-day mortality in critically ill patients [24]. This prospective study of shock patients using the data extracted from West China Hospital database, the largest hospital in Western China, analyzes the epidemic characteristics of hemodynamics and pulmonary pathology assessed by CCUS within the initial 6 hours after shock onset and whether specific ultrasonic variables carry potential value in predicting patient outcome.

## 2. Methods

### 2.1. Ethics Approval and Consent to Participate

The study was approved by the Ethics Committee of West China Hospital Review Board for Human Research with the following reference number 201736. All patients or families gave their informed consent to participate.

### 2.2. Study Design

#### 2.2.1. Selection and Description of Participants

This prospective study of shock patients in CCUS was conducted in 181 adult patients admitted between April 2016 and June 2017 in West China Hospital.

The adult patients admitted with shock who met the following shock criteria [1–3] were screened by intensivists. Those who were included and obtained CCUS exam within the initial 6 hours were those who met the following criteria: (1) aged 18 years or older, (2) state of hypotension with systolic arterial pressure below 90 mmHg, mean arterial pressure below 70 mmHg, or a reduction in systolic blood pressure more than 40 mmHg from baseline, (3) hyperlactatemia (arterial lactate above 2 mmol per liter), oliguria (urine output < 0.5 ml/kg/hr), and (4) at first 6 hours from the onset of shock. Patients were excluded if they met one of the following criteria: (1) age below 18 years, (2) pregnant, and (3) patients or families refused to be enrolled.

### 2.3. CCUS Exam Protocol and Data Collection

**Figure 1:**
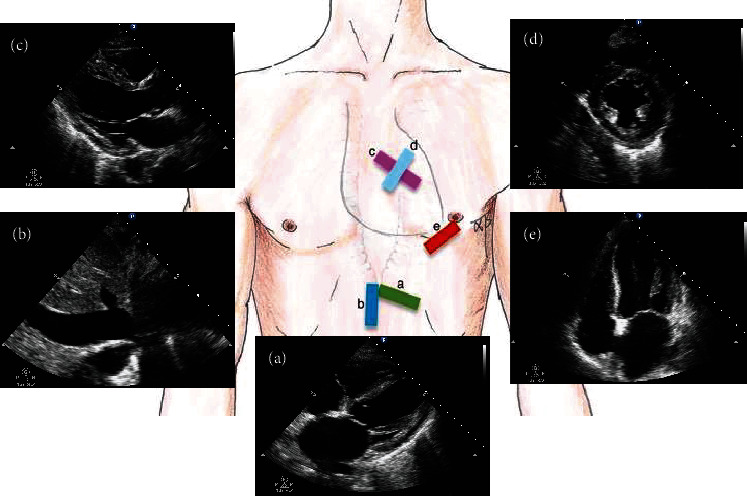
Five standard views used in critical care ultrasonography (CCUS). Subxiphoid long-axis (SLAX) view (a), subxiphoid inferior vena cava (SIVC) view (b), parasternal long-axis (PLAX) view (c), parasternal short-axis (PSAX) view (d), and apical four-chamber (A4CH) view (e).

Based on the critical care chest ultrasonic examination (CCUE) protocol [25, 26], five standard views of the Echo (Figure 1) and the measurement of each view (Table 1) were recorded for the shock patients obtaining CCUS 6 hours after admission. CCUS examinations were performed by a board-certified physician who has completed a full CCUS training course with more than 6 months of experience in critical care ultrasonic practice. Meanwhile, the results which categorized images as “normal” or “abnormal” had been reviewed by senior physicians. The ultrasound instrument (Philips CX50, Washington, USA, and Sonosite M-Turbo, Washington, USA) had an ordinary convex probe and an array probe that was used for the data collection. Ultrasound examination findings and clinical data were collected in a standardized recorded form. The investigators recorded the ultrasonic data which was blinded to the treatment team and followed the outcome. The data consisting of clinical and ultrasonic variables were entered into the database after patient's discharge or death.

Save 4-6 s for each ultrasound video, then use the data collected to analyze cardiac structural abnormalities, volume status, responsiveness, and systolic and diastolic dysfunction. Defined terms for cardiac structural abnormalities include the measured areas of the right ventricle (RV) and left ventricle (LV). For instance, RV dilation is defined as RV : LV area ratio > 0.6 on apical four-chamber view (A4CH) [17, 27, 28]. LV dilation is left ventricular end-diastolic dimension (LVEDD) > 5 cm on parasternal long-axis (PLAX) view, and ventricular hypertrophy is defined when the interventricular septum (IVS) or LV posterior wall thickness is >1.1 cm at end-diastolic from the PLAX view [29, 30]. The extent of the pericardial effusion, which was semiquantitatively assessed in subxiphoid long-axis (SLAX) view, is defined by the following terms [17, 24, 31–33]: “small,” pericardial effusion typically resides in the posterior groove with depth < 1 cm and only in systole; “moderate,” effusion will reside in the posterior groove ± everywhere and a depth of 1-2 cm; and “large,” effusion will be circumferential and >2 cm in depth. Volume status or volume responsiveness [23, 34–38] is calculated by using the diameter and distensibility index of the inferior vena cava (dIVC) during controlled ventilation. If the diameter of IVC less than 1 cm, or the diamter of IVC is 1-2 cm and the dIVC > 18% which indicated hypovolemia. And the diameter of IVC more than 2 cm, or the dIVC < 18% while the diameter of IVC is 1-2 cm which indicated hypervolume. Evaluation of RV systolic function is indicated with a TAPSE < 1.7 cm or S′‐TV < 9.5 cm/s [29, 39, 40]. LV systolic function [29, 41–44] is assessed using MAPSE (<1.2 cm) and S′‐MV < 8 cm/s to suggest LV dysfunction subcategorized using EF classified into normal (EF > 55%), mild (EF 45-54%), moderate (EF 30-44%), and severe dysfunction (EF < 30%). LV diastolic function [45–47] is defined by the EAE/ASE recommendations which were recently updated in 2016 [45].

**Figure 2:**
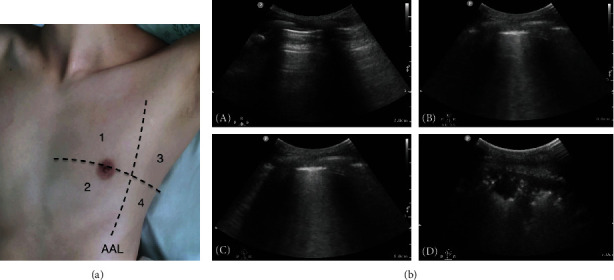
Eight-zone lung ultrasound examination protocol and lung ultrasound pattern. (a) Each hemithorax is separated into four quadrants: anterior, lateral zones (separated by the anterior axillary lines) each divided into upper and lower portions. AAL indicates anterior axillary line. (b) Lung ultrasound pattern: (A) A pattern; (B) B1 pattern; (C) B2 pattern; (D) C pattern (lung consolidation) [52].

In order to analyze shock patients' lung pathophysiological changes, we used eight-zone lung ultrasound (LUS) examination protocol according to the international evidence-based recommendations for point-of-care lung ultrasound [11, 25, 26] (Figure 2). Anterior lateral zones (separated by the anterior axillary lines) are each divided into upper and lower portions of the right and left lung. The LUS exam was required to identify lung sliding, lung point, A lines, B lines, consolidation/atelectasis, and pleural effusion. Scoring LUS patterns in each exam region was based on the following criteria: 0 point for the presence of lung sliding with A lines or fewer than two isolated B lines; 1 point for multiple, well-defined B lines (B1 lines); 2 points for multiple coalescent B lines (B2 lines); and 3 points for the presence of lung consolidation. Ultrasound patterns worth the highest points were recorded in each zone, and a sum total was calculated with the max possible score of 24 [11, 48–51].

The shock patients' demographic and clinical characteristics and the ultrasonic pattern of hemodynamics were documented as part of data analysis after completion of the study. A stepwise bivariate logistic regression model was established to identify the correlation between the ultrasonic variables and the 28-day mortality of shock patients.

### 2.4. Statistical Analysis

All statistical analyses were performed using SPSS 24.0 statistical software. Continuous data were reported as the mean ± SD for parametric data and the median with interquartile range (IQR) for nonparametric data or as counts and percentages for categorical variables. Univariate regression analysis was used to define significant relations between the ultrasonic variables of cardiorespiratory and 28-day mortality. The multivariate analysis was conducted to determine whether the ultrasonic variables of cardiorespiratory were independently related to 28-day mortality. *p* value < 0.05 was considered to be statistically significant.

## 3. Results

### 3.1. Demographic and Clinical Characteristics

The study was comprised of 181 shock patients (113 men and 68 women) with a mean age of 58.2 ± 18.0 years during April 2016 to June 2017. The mean heart rate (HR) was 117.1 ± 24.4 beats per minute, and the average mean arterial pressure (MAP) was 79.3 ± 15.4 mmHg on admission. 162 (89.5%) and 16 (8.8%) shock patients required a vasopressor and inotrope infusion to maintain MAP > 65 mmHg. The median lactate was 3.2 ((interquartile range (IQR), 2.0-6.8)), median urine output per hour was 50 ml (IQR, 20-90), and average APACHE II score was 23.7 ± 8.7. 179 patients (98.9%) were mechanically ventilated, the median time on ventilator support was 168 hours (IQR, 94-384), and median PaO_2_/FiO_2_ was 185 (IQR, 124.9-266.2). Among the four subtypes of shock (distributive, hypovolemic, cardiogenic, and obstructive), distributive shock was considered to be the most common [*n* = 111 (61.3%)], followed by hypovolemic shock [*n* = 54 (29.8%)], cardiogenic shock [*n* = 12 (6.6%)], and obstructive shock [*n* = 4 (2.2%)]. The median length of ICU and hospital stay was 15 days (IQR, 7-28) and 24 days (IQR, 13-38), respectively. 28-day mortality resulted in 44.8% (*n* = 81) (Table 2). The discharge diagnosis of all shock patients is presented in Table 3.

### 3.2. Ultrasonic Pattern of Hemodynamics of the Cases of Shock Patients

**Figure 3:**
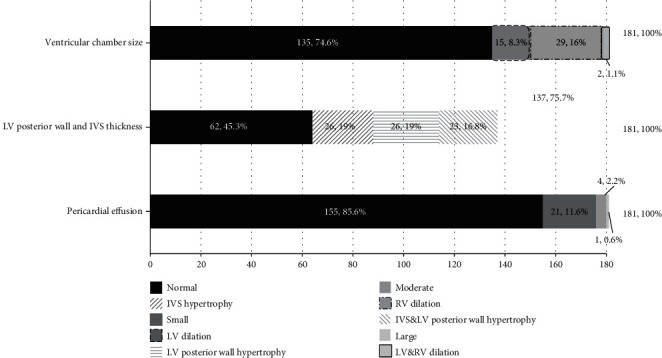
Completion rate and findings of cardiac structure and pericardial effusion in shock patients. Abbreviations: LV: left ventricle; RV: right ventricle; IVS: interventricular septum.

The results of the initial CCUS conducted during the first six hours from shock onset are summarized in Table 4. The results of the cardiac assessment included 31 cases with RV dilation, 17 with LV dilation, and 75 patients with IVS or LV posterior hypertrophy (Figure 3). 26 patients had pericardial effusions with a mean diameter of 0.997 ± 0.34 cm.

**Figure 4:**
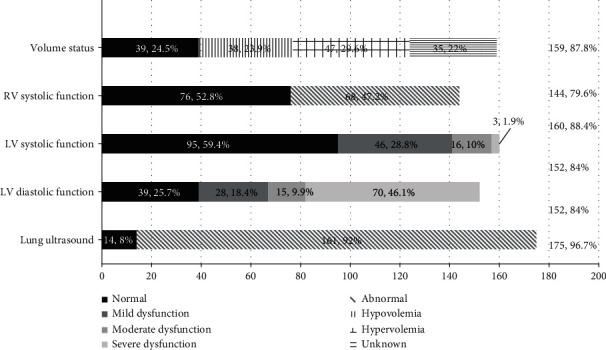
Completion rate and findings of cardiac function, volume status, and lung ultrasound on shock patients. Abbreviations: LV: left ventricle; RV: right ventricle; LA: left atrium.

159 of 181 (87.8%) cases received the IVC exam. 124 cases had definite volume status by IVC examination (78.0%) consisting of hypovolemia in 38 (23.9%) cases, intermediate status volume found in 39 (24.5%) cases, hypervolemia in 47 (29.6%) cases, and 35 (22%) cases that did not fulfill the criterion to assess the volume status by IVC examination (Figure 4).

TAPSE was measured in 143 of 181 (79%) cases, and S ′-TV was measured in 129 (71.3%) cases for evaluating RV function. The mean TAPSE was 1.76 ± 0.53 cm, and mean S′-TV was 15.88 ± 5.71 cm/s. RV dysfunction was found in 68 cases (47.2%) (Figure 4).

LV systolic function was evaluated in 160 (88.4%) cases, and of these cases, EF measurement was calculated in 111 cases, and MAPSE and S′-MV measured in 146 cases. LV dysfunction was found in 65 (40.6%) cases, in which the mild, moderate, and severe dysfunction was 46 (28.8%), 16 (10%), and 3 (1.9%), respectively (Figure 4).

152 of 181 cases (84%) received LV diastolic function evaluation. Of those cases, 113 (74.3%) cases were labeled as “abnormal,” in which the mild, moderate, and severe dysfunction was 28 (18.4%), 15 (9.9%), and 70 (46.1%) cases, respectively (Figure 4).

**Figure 5:**
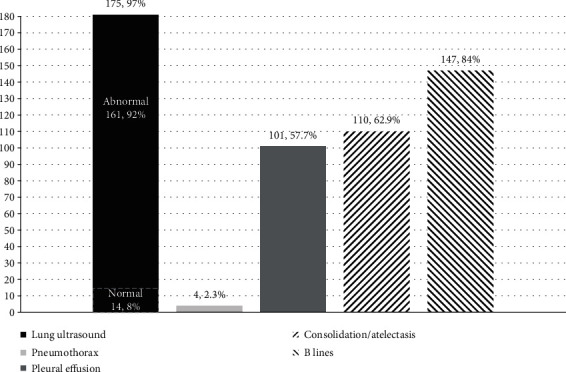
Completion rate and findings of lung ultrasound examination on shock patients.

Lung ultrasound exam has been done in 175 (96.7%) out of 181 patients. Of those examined, positive lung pathology changes were found in 161 cases (92.0%); the abnormal findings were listed as pneumothorax in 4 (2.3%) cases, pleural effusion in 101 (57.7%) cases, consolidation/atelectasis in 110 (62.9%) cases, and B lines detected in 147 (84%) cases (Figure 5).

### 3.3. Prognosis Analysis

The ultrasonic variables of volume status, RV and LV systolic function, LV diastolic function, and LUS exam of shock patients are shown in Table 4. These variables were assessed in univariate correlation analysis, which revealed that 28-day mortality was correlated with MAPSE, E/e′, LUSS, abnormal volume status, LV systolic dysfunction, and elevated E/e′ (*p* = 0.032, 0.002, 0.001, 0.038, 0.011, and 0.01, respectively) (Table 5).

The variables in the multivariate analysis of clinical concerns include dIVC, EF, MAPSE, S′-MV, E/e′, TAPSE, S′-TV, LUSS, age, HR, MAP, lactate, urine output per hour, PaO_2_/FiO_2_, and APACHE II. Among the data analyzed, lactate, PaO_2_/FiO_2_, APACHE II, MAPSE, S′-MV, TAPSE, and LUSS were the independent risk factors for 28-day mortality, as shown in Table 6.

## 4. Discussion

The significance of the study exhibited global hemodynamic characteristics and changes with encompassing clinical and ultrasound examination during the early stages of shock in 181 shock patients. This study demonstrated that abnormal expression of cardiac structure in most shock patients (25.4% with ventricular dilation, 54.7% with myocardial hypertrophy) may lead to the inaccurate monitoring of parameters and therefore misguiding the therapeutic strategy [5, 8, 16]. In addition, 74.3% (113/152) cases were diagnosed with a LV diastolic dysfunction; a crucial finding that could not otherwise be detected by conventional monitoring devices [5–7, 10]. The multivariate analysis of the variables has shown that MAPSE, S′-MV, TAPSE, LUSS, APACHE II, lactate, and PaO_2_/FiO_2_ were independently related with 28-day mortality, MAPSE and S′-MV responding best for the left heart function, and TAPSE for the right heart function and LUSS lung pathological changes [11, 29, 39, 41–44].

Our results support our hypothesis that CCUS carries significant advantages in hemodynamic monitoring and therapeutic decision-making towards shock patients [11–17, 21]. First, we believed that CCUS is able to be readily utilized for visualizing hemodynamic changes that may otherwise jeopardize the accuracy of monitoring parameters and therapeutic decision-making strategy. Second, the RV dysfunction and LV diastolic dysfunction, also critical in directing medical management, could be identified rapidly and accurately by CCUS, irreplaceable by other investigative bedside tools. Third, as mentioned earlier, we have obtained comprehensive results proving that MAPSE, S′-MV, TAPSE, and LUSS are independently related with 28-day mortality, indicating that the ultrasound variables can be regarded as evaluation markers just as the well-used indexes such as lactate and APACHE II. Finally, our study provides a detailed framework that systematically describes the hemodynamic characteristics and pathophysiology changes in shock patients with integrated information of lung injury, providing relevant background information in CCUS guiding to accurate treatment.

Although transthoracic echocardiogram is the most commonly performed cardiac ultrasound examination, the disadvantages still cannot be ignored [5, 9]. For example, patient-related conditions (i.e., adiposity, COPD, digestive gas, abdominal compartment syndrome, middle abdominal incision, and chest tube) can strongly impact the image quality of ultrasound examination. Under these circumstances, a transesophageal echocardiograph (TEE) is necessary as auxiliary examination [40, 53, 54]. In spite of this, we still achieved a high completion rate during the CCUS examinations. All patients received structure of chamber size and pericardial effusion exam; the diameter of IVS and LV posterior wall was measured in 75.7% of cases, IVC was measured in 87.8% of cases to evaluate volume status, and those that received evaluation for RV systolic function, LV systolic function, and LV diastolic function were 79.6%, 88.4%, and 84%, respectively. 96.7% of cases received lung ultrasound exam.

As the data has shown in Figures 3 and 4, we detected 54.7% of ventricular wall hypertrophy and 74.3% of diastolic dysfunction. The underlying mechanism might be that severe hypovolemic shock can lead to normal myocardial hypertrophy and myocardium thickening [17] and, in addition, may demonstrate a higher incidence of diastolic dysfunction [46]. These findings add substantially for our physicians to arrive at an accurate diagnosis and thereby implement an appropriate therapeutic plan [17, 40].

The significance extracted from LUS provides a powerful methodology for lung pathological changes that add more valuable information compared to a single chest X-ray exam [11, 25, 26]. Figures 4 and 5 demonstrate that 92% of cases of the patients who received LUS exam had abnormal findings, with which we have not only identified pneumothorax (2.3%), pleural effusion (57.7%), consolidation/atelectasis (62.9%), and B lines (84%) more accurately and rapidly but also measured the lung fluid status semiquantitatively with LUSS as well as discovered the distribution visually [49, 55, 56]. These findings also comply with the results of our previous study [24, 56].

We are aware that our research may have limitations. Despite this, we believe our work could be a catalyst towards making accurate diagnosis directing appropriate treatment for shock patients. In this observational study, it was difficult to determine whether the cardiac dysfunction in shock patients was the cause or the result of shock. Consequently, the prognosis value of MAPSE, S′-MV, TAPSE, and LUSS still requires further randomized controlled trials to explore their role. However, the primary purpose of our study is to describe characteristics of ultrasonic hemodynamic pattern, rather than research the prognosis of shock. In addition, the shock patients' population came from the end point of the referral center for the whole west part of China. This might affect the representativeness of the study to generally healthier patient population.

There is a widespread belief that ultrasound is an operator-dependent technique. In our study, the operator responsible for CCUS assessment was selected as someone who had completed a full CCUS training course and had more than a half-year experience of critical care ultrasound performance. Moreover, all the CCUS assessments were done within 30 minutes and diagnostic results deemed as “normal” or “abnormal” images were double-checked by other senior physicians. Despite the limitations noted above, this study has provided a significant sample of relevant information about the cardiorespiratory characteristic of shock patients assessed by ultrasound exam and may be valuable for the clinical decision-making and subsequent design of clinical trials related to CCUS.

## 5. Conclusions

In conclusion, based on our study, CCUS exam on shock patients performed by experienced physicians can provide valuable detailed findings not otherwise offered by other monitoring devices. Moreover, LV dysfunction, RV dysfunction, and LUSS are independently related to patients' outcome. These results show that CCUS may play a crucial role in patients' assessment and help the physician have a great understanding of its hemodynamic characteristics and involved lung pathology. A well-designed prospective cohort study should be conducted to verify the above results.

## Figures and Tables

**Table 1:** Echocardiography exam protocol and data.

Echocardiography views	Assessment
SLAX	Extent of the pericardial effusion and diameter
SIVC	Diameter of IVC; dIVC = (IVC_max_ − IVC_min_)/IVC_min_
PLAX	IVS and LV posterior thickness LVEDD at end diastolic
PSAX	Eyeballing evaluate LV and RV function and structure
A4CH	M-model: MAPSE, TAPSE TDI: S′-MV, S′-TV RV: LV area ratio at end diastolic Color Doppler: E-MV, A-MV E/e′

Abbreviations: SLAX: subxiphoid long axis; SIVC: subxiphoid inferior vena cava; PLAX: parasternal long axis; PSAX: parasternal short axis; A4CH: apical four chamber; IVC: inferior vena cava; dIVC: distensibility index of the inferior vena cava; IVS: interventricular septum; LVEDD: left ventricular end-diastolic dimension; TAPSE: tricuspid annular plane systolic excursion; EF: ejection fraction; MAPSE: mitral annular plane systolic excursion; E-MV: early diastolic transmitral velocity; A-MV: late diastolic transmitral velocity; TDI: tissue Doppler imaging; S′-TV: tricuspid annular peak systolic velocity; S′-MV: mitral annular peak systolic velocity; E/e′: early diastolic transmitral velocity to early mitral annulus diastolic velocity ratio; RV: right ventricle; LV: left ventricle; TV: tricuspid valve; MV: mitral valve.

**Table 2:** Demographic and clinical characteristics of shock patients and outcome of the studied subjects.

Variable	Measure	Range
Gender (male/female)	113/68	—
Age (years)	58.2 ± 18.0	20-89
APACHE II	23.7 ± 8.7	2-50
Heart rate (bpm)	117.1 ± 24.4	65-180
Systolic blood pressure (mmHg)	111.9 ± 20.9	59-174
Diastolic blood pressure (mmHg)	63.1 ± 15.0	32-132
Mean blood pressure (mmHg)	79.3 ± 15.4	43.7-136.3
Urine output per hour (ml)	50 (20, 90)	0-500
Lactate (mmol/l)	3.2 (2.0, 6.8)	1-28.2
Length of mechanical ventilation (hours)	168 (94, 384)	5-1405
PaO_2_/FiO_2_	185 (124.9, 266.2)	44-620
Type of shock/case (%)		
Distributive shock	111 (61.3)	111/181
Hypovolemic shock	54 (29.8)	54/181
Cardiogenic shock	12 (6.6)	12/181
Obstructive shock	4 (2.2)	4/181
Vasoactive agents/case (%)	162 (89.5)	162/181
Norepinephrine/case (%)	155 (95.7)	155/162
<0.5 *μ*g/kg·min	82 (50.6)	82/162
0.5-1 *μ*g/kg·min	35 (21.6)	35/162
>1 *μ*g/kg·min	38 (23.5)	38/162
Dopamine/case (%)	7 (4.3)	7/162
Inotrope agents/case (%)	16 (8.8)	16/181
ICU length of stay (d)	15 (7, 28)	2-138
Hospital length of stay (d)	24 (13, 38)	2-149
28-day mortality (%)	44.8	81/181

Values were expressed as mean ± standard deviation or median (interquartile range), according to the type of data and data distribution. Percentages for categorical variables and continuous variables were expressed as ranges. Abbreviations: APACHE II: Acute Physiology and Chronic Health Evaluation II; ICU: intensive care unit.

**Table 3:** The discharge diagnoses and the proportion.

Diagnosis	**n** = 181	%
Respiratory disease	37	20.44%
Severe pneumonia	21	11.60%
AECOPD	6	3.31%
ARDS	8	4.42%
Tracheoesophageal fistula	2	1.10%
Abdominal diseases	52	28.73%
Severe acute pancreatitis	24	13.26%
Gastrointestinal perforation	13	7.18%
Bowel obstruction	6	3.31%
Tumor	3	1.66%
Acute obstructive suppurative cholangitis	6	3.31%
Bloodstream infection	5	2.76%
Subcutaneous infection	5	2.76%
Urinary tract infection	4	2.21%
CNS infection	3	1.66%
Intestinal infection	3	1.66%
Infective endocarditis	1	0.55%
Gastrointestinal bleeding	21	11.60%
Arterial aneurysm	4	2.21%
Multiple trauma	2	1.10%
Cardiac arrest	14	7.73%
Heart failure (acute/chronic)	2	1.10%
Myocardial infarction	3	1.66%
Malignant arrhythmia	1	0.55%
High-level spinal cord injury	2	1.10%
Pulmonary embolism	3	1.66%
Pericardial tamponade	1	0.55%
Stroke	11	6.08%
Organ transplantation	7	3.87%

Abbreviations: AECOPD: acute exacerbation of a chronic obstructive pulmonary disease; ARDS: acute respiratory distress syndrome; CNS: central nervous system.

**Table 4:** Cardiorespiratory ultrasonic variables of shock patients.

Cardiac structure/case (%)	Measure	Range
Ventricular chamber size abnormal		
RV : LV area ratio	0.59 ± 0.37	(0.18, 3.0)
RV dilatation/case (%)	31.0 (17.1)	
LVEDD (cm)	4.24 ± 0.65	(2.84, 6.05)
LV dilatation/case (%)	17.0 (13.0)	
Ventricular wall hypertrophic/case (%)	75.0 (54.7)	
IVS (cm)	1.03 ± 0.28	(0.5, 1.92)
LV posterior wall (cm)	1.05 ± 0.32	(0.57, 2.86)
Pericardial effusion/case (%)	26.0 (14.4)	
Volume of pericardial effusion (cm)	0.997 ± 0.34	(0.6, 2.2)
Diameter of pericardial effusion/case (%)		
<1 cm	21.0 (11.6)	
1-2 cm	4.0 (2.2)	
>2 cm	1.0 (0.6)	
Diameter of IVC (cm)	1.71 ± 0.45	(0.55, 2.73)
dIVC (%)	10.77 (4.23, 33.89)	(0, 480.91)
Intermediate status volume/case (%)	39.0 (24.5)	
IVC abnormal/case (%)	85.0 (53.5)	
Hypovolemia	38.0 (23.9)	
Hypervolemia	47.0 (29.6)	
Unknown/case (%)	35.0 (22.0)	
RV dysfunction/case (%)	68.0 (47.2)	
TAPSE (cm)	1.76 ± 0.53	(0.77, 3.48)
S′-TV (cm/s)	15.88 ± 5.71	(3.04, 32.3)
LV systolic dysfunction/case (%)	65.0 (40.6)	
EF (%)	56.93 ± 12.35	(16.9, 88)
MAPSE (cm)	1.23 ± 0.42	(0.27, 2.36)
S′-MV (cm/s)	12.20 ± 4.29	(2.71, 24.0)
LV diastolic dysfunction/case (%)	113.0 (74.3)	
MV-E	94.07 ± 33.66	(37.20, 253.0)
MV-A	82.78 ± 28.67	(32.4, 188.0)
MV-E/A	1.07 ± 0.46	(0.52, 2.70)
MV-e′	12.04 ± 6.49	(1.55, 62.80)
MV-a′	11.59 ± 4.07	(2.09, 26.90)
E/e′	9.56 ± 4.18	(3.22, 29.62)
LUSS	9.47 ± 5.89	(0, 22.0)
LUS abnormal/case (%)	161.0 (92.0)	
Pneumothorax/case (%)	4.0 (2.3)	
Pleural effusion/case (%)	101.0 (57.7)	
Consolidation/atelectasis/case (%)	110.0 (62.9)	
B lines/case (%)	147.0 (84.0)	

Values were expressed as mean ± standard deviation or median (interquartile range), according to the type of data and data distribution. Abbreviations: RV: right ventricle; LV: left ventricle; IVS: interventricular septum; LVEDD: left ventricular end-diastolic dimension; IVC: inferior vena cava; dIVC: distensibility index of the inferior vena cava; TAPSE: tricuspid annular plane systolic excursion; EF: ejection fraction; MAPSE: mitral annular plane systolic excursion; MV: mitral valve; MV-E: early diastolic transmitral velocity; MV-A: late diastolic transmitral velocity; e′: early mitral annulus diastolic velocity; a′: later mitral annulus diastolic velocity; S′-TV: tricuspid annular peak systolic velocity; S′-MV: mitral annular peak systolic velocity; E/e′: early diastolic transmitral velocity to early mitral annulus diastolic velocity ratio; LUS: lung ultrasound; LUSS: lung ultrasound score.

**Table 5:** Univariate correlation analysis: regression coefficients (*r*) and *p* values.

Indexes	28-day mortality		
	*r*	*p*	95% CI
Age	0.021	0.017	1.004-1.038
HR	0.014	0.029	1.001-1.027
MAP	-0.004	0.694	0.997-1.015
APACHE II	0.088	<0.010	1.049-1.137
Lactate	0.162	<0.010	1.089-1.270
Urine output per hour	-0.008	0.010	0.987-0.998
Vasoactive agents	1.223	0.036	1.081-10.677
PaO_2_/FiO_2_	-0.003	0.031	0.994-1.000
Diameter of IVC	0.029	0.935	0.515-2.056
dIVC	-0.016	0.089	0.966-1.002
EF	-0.024	0.132	0.946-1.007
MAPSE	-0.894	0.032	0.181-0.926
S′-MV	-0.020	0.611	0.908-1.058
TAPSE	-0.607	0.066	0.286-1.041
S′-TV	-0.006	0.854	0.946-1.069
E/e′	0.169	0.002	1.063-1.321
LUSS	0.091	0.001	1.037-1.156
Ventricular dilation	0.401	0.242	0.763-2.923
Ventricular hypertrophy	0.221	0.521	0.635-2.448
Abnormal volume status	-0.818	0.038	0.204-0.956
LV systolic dysfunction	0.836	0.011	1.211-4.393
LV diastolic dysfunction	0.452	0.233	0.748-3.305
RV systolic dysfunction	0.609	0.071	0.948-3.565
Elevated E/e′	1.720	0.010	1.512-20.622

Abbreviations: HR: heart rate; MAP: mean arterial pressure; APACHE II: Acute Physiology and Chronic Health Evaluation II; IVC: inferior vena cava; dIVC: distensibility index of the inferior vena cava; EF: ejection fraction; MAPSE: mitral annular plane systolic excursion; S′-MV: mitral annular peak systolic velocity; TAPSE: tricuspid annular plane systolic excursion; S′-TV: tricuspid annular peak systolic velocity; E/e′: early diastolic transmitral velocity to early mitral annulus diastolic velocity ratio; LUSS: lung ultrasound score; LV: left ventricle; RV: right ventricle.

**Table 6:** Multivariate analysis between the cardiorespiratory ultrasonic variables and clinical indexes and 28-day mortality.

Indexes	28-day mortality		
	OR	*p*	95% CI
Age	1.020	0.462	0.967-1.077
HR	0.979	0.430	0.930-1.031
MAP	0.989	0.636	0.945-1.035
Lactate	1.324	0.010	1.069-1.640
Urine output per hour	0.992	0.305	0.976-1.008
PaO_2_/FiO_2_	1.011	0.028	1.001-1.022
APACHE II	1.132	0.027	1.014-1.263
dIVC	0.970	0.152	0.930-1.011
EF	0.950	0.306	0.862-1.048
MAPSE	0.032	0.047	0.001-0.959
S′-MV	1.379	0.041	1.013-1.879
E/e′	1.277	0.172	0.899-1.814
TAPSE	0.066	0.022	0.006-0.681
S′-TV	1.337	0.052	0.998-1.791
LUSS	1.383	0.002	1.122-1.704

Abbreviations: HR: heart rate; MAP: mean arterial pressure; APACHE II: Acute Physiology and Chronic Health Evaluation II; dIVC: distensibility index of the inferior vena cava; EF: ejection fraction; MAPSE: mitral annular plane systolic excursion; S′-MV: mitral annular peak systolic velocity; E/e′: early diastolic transmitral velocity to early mitral annulus diastolic velocity ratio; TAPSE: tricuspid annular plane systolic excursion; S′-TV: tricuspid annular peak systolic velocity; LUSS: lung ultrasound score.

## Data Availability

The data used to support the findings of this study are included within the article.
